# Preservation of ancestral Cretaceous microflora recovered from a hypersaline oil reservoir

**DOI:** 10.1038/srep22960

**Published:** 2016-03-11

**Authors:** Grégoire Gales, Nicolas Tsesmetzis, Isabel Neria, Didier Alazard, Stéphanie Coulon, Bart P. Lomans, Dominique Morin, Bernard Ollivier, Jean Borgomano, Catherine Joulian

**Affiliations:** 1 Aix-Marseille Université, CEREGE, Centre St Charles, Case 67, 3 Place Victor Hugo, 13331 Marseille, France; 2 Aix-Marseille Université, Université du Sud Toulon-Var, CNRS/INSU, IRD, MIO, UM 110, 13288, Marseille, cedex 09, France; 3 Shell International Exploration and Production Inc., 3333 Highway 6 South, Houston, Texas 77082, USA; 4 BRGM, Unité BioGéochimie Environnementale, 3 Avenue Claude Guillemin, BP 36009, 45060 ORLEANS cedex 2, France; 5 Emerging Technologies – Subsurface, Projects & Technologies, Shell Global Solutions International B.V., Kessler Park 1, 2288 GS Rijswijk, The Netherlands

## Abstract

Microbiology of a hypersaline oil reservoir located in Central Africa was investigated with molecular and culture methods applied to preserved core samples. Here we show that the community structure was partially acquired during sedimentation, as many prokaryotic 16S rRNA gene sequences retrieved from the extracted DNA are phylogenetically related to actual *Archaea* inhabiting surface evaporitic environments, similar to the Cretaceous sediment paleoenvironment. Results are discussed in term of microorganisms and/or DNA preservation in such hypersaline and Mg-rich solutions. High salt concentrations together with anaerobic conditions could have preserved microbial/molecular diversity originating from the ancient sediment basin wherein organic matter was deposited.

Subsurface environments harbor 1/10 to 1/3 of global living biomass, thus playing an important role in biogeochemical cycling of elements^1^,^2^. Subsurface oil-bearing reservoir rocks have been extensively studied during the last century with respect to the critical economic value of hydrocarbons. The probable rarefaction of this fossil resource and the possibility to sequestrate CO_2_ within depleted oil fields^3^ stimulate multidisciplinary scientific investigations including microbiology. Whereas the presence of microorganisms in oil reservoirs was recognized earlier^4^, the reality of their effective *in situ* activity remains elusive^1^. Biodegradation of organic matter in sedimentary rocks contributes to the biogeochemical cycling of carbon^1^ and strongly impacts the quality and exploitation of hydrocarbons^1^. Oil reservoirs are nutrient-depleted environments, especially lacking of phosphorus and nitrogen. They contain an excess of reduced electron donors (hydrocarbons) but a shortage of electron acceptors (*e.g.*, nitrate, oxygen…) and are considered as anaerobic environments^5^,^6^,^7^. We characterized the indigenous microbiota of a hypersaline oil reservoir by molecular and cultural analyzes of rock samples, rather than from fluid samples (*e.g.* production fluid from well heads of oil producing wells) possibly affected by greater exogenous microbial contamination^8^. The core was collected from an onshore oil reservoir in Central Africa, before oil exploitation and any fluid injection.

## Results

### Core chemistry and mineralogy

**Figure 1:**
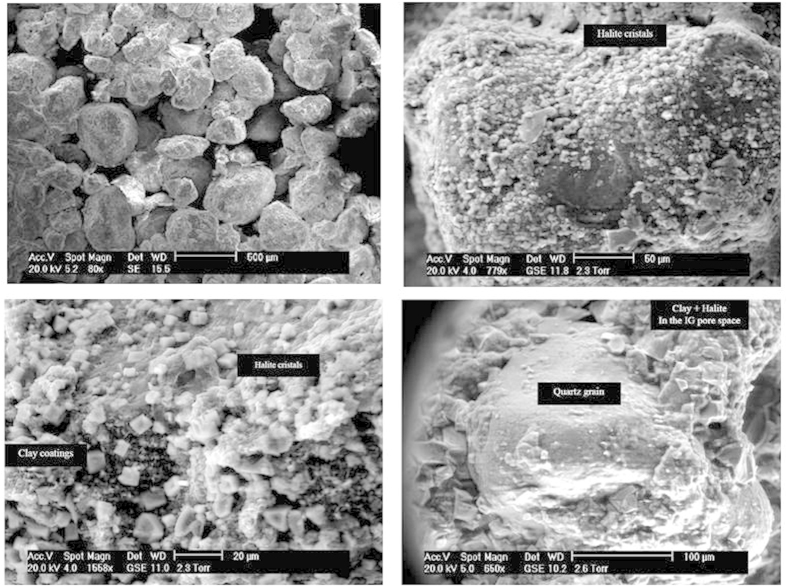
SEM image of the sand. Quartz-felspar grains are coated with clays, salt (NaCl) crystal crystalized on the grain surfaces.

The core was sampled at 1153–1154 m depth in a lower Cretaceous sandstone, underlying the transgressive Gamba Formation and thick salt deposits^9^. Reservoir pressure and temperature were respectively 12 Mpa and 43 °C. These sediments are associated to the South Atlantic Aptian salt deposition contemporary of the basin creation resulting from the break-up between Africa and South America^10^. The unconsolidated sandstone consisted in quartz-felspar grains coated with green clays and salt crystals, without stratification. NaCl crystals were very abundant on the grain surfaces and within the clay coatings (Fig. 1). Intergranular cements, such as quartz, were absent. Most of the grains were coated by clays (smectite, chlorite, illite), which probably inhibited the development of quartz overgrowth in the intergranular pore space. Local chemical analyzes and X-ray diffraction on the bulk sample and on separated clays confirmed the nature of the minerals. Moreover, as chlorite minerals from the inner core were not oxidized, anaerobic conditions during coring, sample preservation (by aluminium barrel, freezing in liquid nitrogen), shipping to and transferring at the laboratory should have been preserved. In this respect, our sampling conditions should not have affected the composition of the existing microbial diversity of the inner section (6 cm) of the 10 cm core. Chemical analyzes are summarized in Table S1. Formation waters had salinity close to saturation and the core itself contained above 13 mg/g of Na^+^ (supplementary material, Table S1).

### Bacterial and archaeal molecular diversities

16S rRNA gene surveys, including classical clone libraries analyses (case of *Bacteria* and *Archaea*) and high throughput sequencing methods (case of *Bacteria*) were conducted on DNA extracted directly from the core (Tables 1 and 2). The rarefaction curves from cloning (supplementary material Fig. S2) showed that the Rabi core harbored low archaeal diversity as saturation was obtained. But the Rabi core was inhabited by a more diverse bacterial community than the one estimated by the clone library as the rarefaction curve was not saturated, highlighting the need to perform deeper 16S rRNA gene sequencing to assess it.

### Clone libraries analyses

Most of the bacterial OTUs (Operational Taxon Units) were related to aerobes and facultative anaerobes known to degrade oil compounds. From 92 clones analyzed, the library was dominated (50%) by OTU related to aerobic *Ochrobactrum* spp., of which some strains were found in crude oil or reported to degrade aliphatic and aromatic hydrocarbons^11^. Three OTUs (13% of the library) were related to facultative anaerobic, halophilic marine species of the *Halomonas* genus with *H. shengliensis* being reported to degrade crude oil^12^. A fourth *Halomonadaceae* OTU was related to the halophilic strain *Chromohalobacter salexigens*, formerly *Halomonas elongata*. As well, minor OTUs were related to aerobes of the genera *Burkholderia* (4.3% of the library) and *Microbacterium* (1.1% of the library) of which species are capable to aerobically degrade crude oil^13^,^14^.

Besides the *Halomonas* spp., other OTUs were also related to halophilic microbes, a physiological trait compatible with the *in situ* salinity. Indeed, the second most dominant OTU (19.6% of the library) was related to the marine genus *Halolactibacillus* recognized as facultative anaerobic halophiles^15^. We retrieved a member of the *Firmicutes* related to the *Halanaerobium* genus (4.3% of the library); members of this genus have been frequently recovered from oil reservoirs^16^,^17^ and some of them inhabit sebkhas similar to the paleoenvironment of Rabi sandstone^18^. We also report the occurrence of members of the genus *Orenia*, family *Halobacteroidaceae*. Interestingly, few representatives of this family have been retrieved by molecular approaches from oil reservoirs so far^19^. They include phylogenetic relatives of the genus *Orenia* recovered from hot reservoirs^19^ where their *in situ* activity is highly questionable. We found only one clone phylogenetically related to the anaerobic, thermophilic and halophilic genus *Geotoga*, order *Thermotogales*. Presence of members of this order is also recurrent in oil fields^16^.

Within the 36 archaeal clones analyzed, the large majority of OTUs belonged to the *Halobacteriaceae* family regrouping mainly extreme aerobic halophiles. Similar microorganisms have been already isolated from high saline reservoirs^20^. The library is dominated (>30%) by OTUs related to *Halococcus hamelinensis* and *Haloplanus natans* isolated from saline environments, respectively stromatolithes from Shark Bay, Australia^21^ and the Dead Sea^22^. Then, 16.7% of the OTUs belonged to the *Halorhabdus* genus; among the two described species within this genus, *H. utahensis* is a facultatively anaerobic, extremely halophilic archaeon isolated from the Great Salt Lake^23^, whereas *H. tiamatea*, isolated from a deep-sea anoxic basin of the Red Sea, grew under anaerobic or microaerobic conditions^24^. As well, 16.7% of the OTUs were related to the newly described aerobic and extreme halophilic archaeon *Halarchaeum acidiphilum* isolated from solar salt^25^. Beside mesophilic representatives, one OTU was related to the *Thermococcaceae* family only represented by hyperthermophilic microorganisms commonly found in hot and low saline ecosystems, including oil reservoirs^16^,^19^. The phylogenetic divergence (only 77% identity) with known *Thermococcaceae* members clearly showed that it represents a new lineage within this family. Very low diversity of archaeal sequences was assessed by the saturation of the rarefaction curve (Supplementary material Fig. S2).

### Bacterial diversity estimated by high throughput sequencing

The most abundant OTU in the core (Table 2) was related to the marine, halophilic, facultative anaerobe *Halomonas* (27.9%). Some *Halomonas* spp. are reported to be involved in the degradation of crude oil^12^ and others are reported to originate from highly saline environments^26^. Besides *Halomonas*, a high proportion of OTUs was related to the thermophilic sulfate-reducing bacterium *Desulfonauticus*, family *Desulfohalobiaceae* (11%). *Desulfonauticus* spp. were previously isolated from oil production water and deep sea hydrothermal vents^27^,^28^. We have also identified a number of OTUs related to the genus *Pseudomonas* (10.1%) comprising species possibly degrading aromatic hydrocarbons both aerobically and anaerobically. Another genus that matched many of the produced OTUs was the facultative anaerobic, halophilic marine bacterium *Marinobacter* (7.1%) members of which are capable to also degrade petroleum hydrocarbons^29^.

In addition to *Desulfonauticus* mentioned above, we identified in moderate amounts some OTUs that are related to anaerobic mesophilic sulfate and/or sulfur reducers such as *Desulfovibrio (*0.8%), order *Desulfovibrionales* (2.9%), *Pelobacter* (0.7%), as well as to anaerobic thermophiles (*e.g. Thermovirga*, 0.4%). Members of these taxa have been already successfully isolated from petroleum hydrocarbon associated environments^30^,^31^,^32^. Additionally, fermentative bacteria such as those belonging to the genus *Halanaerobium* (0.6%), as already mentioned in the bacterial clone library, and the *Clostridiaceae* family (4.2%) were also found to have representative OTUs in the dataset. *Halanaerobium* has been previously found in formation water samples from an oil reservoir^33^ whereas members of the *Clostridiaceae* family are thought to be using hydrocarbon intermediates to generate organic acid precursors under sulfate, methanogenic or iron reducing conditions^34^,^35^,^36^. Other genera related to the produced OTUs include *Oceanobacillus* (1.4%), *Ralstonia* (0.9%), *Marinilactibacillus* (0.3%) as well as members of the families *Enterobacteriaceae* (6.1%), Clone MSBL8 (2.1%), *Oceanospirillaceae* (1.2%), *Comamonadaceae* (1.1%), *Flavobacteriaceae* (0.4%). Finally, OTUs related to members of the order *Bacteroidales* were also found in the sample at a relative high number (5.7%). The significance of many of the above taxa to the Rabi’s *in situ* reservoir conditions is still unknown as many of them are either still poorly characterized in the literature.

### Culture experimentation

Neither sulfate reducers, nitrate reducers, sulfide oxidizers nor hydrogenotrophic and methylotrophic methanogens were encountered. Only fermentative *Bacteria*, and specially members of the phylum *Firmicutes*, representatives of the *Bacillaceae* and *Halobacteroidaceae* families (Table 3) were cultivated. Strains belonging to the genus *Halanaerocella petrolearia* of the family *Halobacteroidaceae* were isolated; this species is perfectly adapted to this environment as it grew anaerobically at high saline concentrations^37^. However, it was not recovered from the molecular surveys. Other isolates were also related to highly saline shallow or surface environments. It is the case of strains closely related to (i) *Halobacillus trueperi* commonly found in Tunisian sebkhas or chotts^38^, (ii) *Orenia marismortui*, which is described as adapted also to highly saline surface environments and degrading complex organic compounds^39^ and (iii) *Thalassobacillus devorans*, formerly isolated from hypersaline habitats and able to oxidize aromatic compounds^40^.

Within archaeal clone library, mainly hyperhalophilic aerobic *Archaea* and facultative anaerobes appeared to be dominant from molecular studies, although the core was essentially anaerobic. However, despite repeated cultivation efforts, none of these archaeons were isolated thus suggesting that they are not metabolically active in the oil field, most probably because of strict anaerobic conditions prevailing *in situ* and limited access to organic matter restricted to hydrocarbons, together with the lack of suitable electron acceptors possibly used by some of these microorganisms (*e.g.* nitrate, fumarate).

## Discussion

Clone library analysis and Illumina sequencing results gave two different pictures of molecular bacterial diversity. Expected strict anaerobes are found essentially from the Illumina sequencing where they account for a larger part of the diversity (27.6%) than in the clone library (8.7%). But the originality of both results is the abundance of aerobic and facultative anaerobic microorganisms in a strictly anaerobic environment. As expected when considering the number of reads, Illumina sequencing showed a greater diversity than the clone library. The applied protocol included a nested-PCR which, in addition to increase specificity, will also increase sensitivity. Sequences initially poorly amplified may be enriched after the nested-PCR step, resulting in a larger diversity retrieved by sequencing. As DNA used for high throughput sequencing came from the same core but after a longer storage time, we may also infer that Illumina sequencing platform, which amplify shorter fragments (440 bp) than one used for the construction of the clone library (1400 bp), has found target sequences on shorter genomic DNA fragments that have been degraded. A 2-step approach was however necessary to produce amplicons from the Rabi core DNA of sufficient yield and quality for Illumina sequencing. Even if acknowledging the introduction of biases, according to in-silico primer analysis, we estimated a limited reduction of coverage of the *Bacteria* lineages, especially if a one mismatch is allowed between primer and template (data not shown).

There is some recovery between molecular and cultural studies, namely the presence of fermentative *Bacillaceae, Halanaerobiaceae* and *Halobacteroidaceae* in the clone libraries (approximately 30% of the clone sequenced) and the effective culture of some representatives of these families. But we didn’t succeed in the cultivation of members of the *Proteobacteria* (about 70% of the clone library and 60% of the high throughput sequencing results), especially in the case of the *Halomonas* or *Ochrobactrum* genera. As the genus *Ochrobactrum* is solely detected in the clone library and not by the culture-dependent method or the Illumina sequencing, its presence may be due to an external contamination of the core, the genus *Ochrobactrum* being clearly retrieved from shallow environments like soils, followed by a PCR artefact. Furthermore, no *Archaea* was cultivated. Some studies reveal the successful isolation of *Archaea* from old and salty environments^41^,^42^. Isolates belonged to *Halobacteriaceae* and *Haloferacaceae*, as sequences retrieved from Rabi core. However, contrary to these environments, the Rabi core is strictly anaerobic and not suitable to sustain viability of these hyperhalophilic aerobic archaeons. It could also be due to the medium formulations or to specific niches being sampled. Discrepancies between culture-dependent and -independent methods targeting biological diversity are common in microbiological studies^43^,^44^. It could reflect our inability to mimic natural environmental conditions sufficiently to sustain growth, and/or our inability to extract and amplify correctly DNA from natural and industrial environments. The retrieved strict anaerobes as well as the facultative ones show traits compatible with the Rabi environment (ability to develop in the absence of oxygen and at high salinity) and have for some of them already been retrieved from oil reservoirs (*e.g. Halanaerobium*, *Orenia*, *Geotoga*, *Desulfonauticus* spp.). Concerning aerobes, there is increasing evidence of an aerobic community including hydrocarbon degraders being inhabitant of oil reservoirs and oil sand cores, one discussed reason would be the *in situ* availability of oxygen possibly originating from meteoric water^45^,^46^. As suggested in Head *et al.*^45^, a cryptic community using oxygen, possibly provided by water radiolysis may be present in some oil reservoirs. Despite, the *in situ* role of recovered aerobic microbes and especially the hyperhalophilic archaeons in the Rabi core is questionable; it is known that some of them are able to grow anaerobically using nitrate or fumarate as terminal electron acceptors. In this respect, unknown types of anaerobic metabolism to be performed by these microorganisms cannot be excluded.

Are they of ecological significance in this ecosystem or do they reflect the existing biodiversity during the sediment settling in the basin before the process of oil filling, resulting in a long-term dormancy? Two hypotheses could afford for the detection of aerobic microorganisms in an anaerobic, highly saline environment. First, microorganisms are not active and DNA has been preserved over millions of years^47^; second, microbial activity is scarce and community has been preserved in a slow-growing state for millions years^48^.

DNA preservation over geological times has been a matter of debate in the subsurface microbiology^47^,^49^,^50^. Although bacterial DNA recovery from historical samples can lead to full genome sequencing of ancient *Bacteria*^51^,^52^, there are nowadays few studies that report the effectiveness of very ancient (at least prior to the Holocene) microbial DNA recovery^53^,^54^. If fully hydrated DNA spontaneously decays over only hundreds of years^55^, extension of the half-life of intact DNA can be achieved by low temperature, high ionic strength, anoxic conditions and protection from enzymatic degradation^56^,^57^. High salt concentrations could thus be a great factor of biomolecule preservation. The salt-saturated solution filling the reservoir pores may explain the microbial diversity and the possible DNA preservation within the oil reservoir. Presence of physiological concentrations of K^+^ and Mg^2+^ strongly reduce thermal degradation of DNA^56^. Protective effect of salt on biological macromolecules such as tRNA is demonstrated at NaCl concentration between 0.5 and 3 M^57^. Effects of the extremely chaotropic and soluble MgCl_2_ salt have been studied in the deep sea hypersaline lake Discovery (eastern Mediterranean)^58^. MgCl_2_ concentration above 1.26 M inhibited the growth of all microorganisms taken from this environment. MgCl_2_ at high concentrations not only denatures macromolecules, but also preserves the more stable ones, like DNA^58^. Other results confirmed the preservation and the transforming power of DNA when solubilized in this type of brine^59^, thus being prone to PCR amplification and cloning. Highly saline and anaerobic solution like those of the studied reservoir (supplementary material, Table S1) can then be a potential reservoir for ancient DNA. Clays coating the grains could also favour the adsorption of DNA, thus preserving it from hydratation. Interestingly, archaeal isolates from Eocene rock salt were polyploid, with genome copy numbers of 11–14 genomes during exponential growth phase^42^. Abundance of DNA in single archaeal cells in Rabi paleoenvironment could explain preservation of archaeal DNA. This strong hypothesis should get more confirmation.

With regard to the second hypothesis, dormant or slow activity communities have been recovered from tenths to million years in a freshwater sediment^60^ or from a surface isolated lake in Antarctica^48^. Moreover, microbial survival related to fermentative activity rather than sporulation has been proven on an up to half a million years period due to fermentation processes in permafrost environment^61^. Both metabolic and phenotypic features should be taken into consideration to explain the presence of microorganisms retrieved by cultural and molecular methods in the reservoir (*e.g.* sporeformers of the order *Bacillales*). As fermentative Prokaryotes constitute the large majority of the microorganisms detected by each technique, and given the amount of reduced hydrocarbon present in the Rabi reservoir, this hypothesis can be viewed as probable as the first. The most important question in the case of the studied oil reservoir is whether the retrieved archaeal 16S rRNA genes belong to dormant *Archaea* that are part of the non-cultivated microorganisms, or are part to dead microorganisms which have no more chance to be cultivated. Presence of both possibly active microorganisms and microorganisms inherited from the time of the sediment deposition suggests that the microbial diversity integrates separate parts of the reservoir history.

Studies of microbial diversity from a core rather from production fluids (*e.g.* formation water) in oil fields are rare and may not be accurate for comparison with our study, due to the difference in salinity. However, there are few reports on the predominance of aerobic microorganisms in oil reservoirs that are generally inhabited by strict anaerobes including sulfate-reducers and methanogens^6^,^7^. The reservoir rock studied here belongs to a cretaceous formation dominated by evaporitic conditions^9^. Protective presence of salt, adsorption on clays and low water activity could have preserved macromolecules such as DNA^56^,^57^,^59^ and may therefore explain the repeated molecular detection of aerobic hyperhalophilic *Archaea*. In this respect, we believe that molecular analyzes in particular provide a frozen picture of the past microbial community existing in the saline sedimentary basin, which is no more active in the hypersaline reservoir studied. This hypothesis is strengthened by the presence of archaeal DNA sequences that are phylogenetically related to those retrieved from actual hypersaline ecosystems, but also from cultivated haloarchaea originating from similar extreme environments. In addition, the anaerobic halophilic bacterium that we have isolated pertains to the family *Halobacteroidaceae* (*e.g. Halobacteroides* spp.), which are common inhabitants of terrestrial saline ecosystems. In contrast, there are several examples of isolation from oil reservoirs in literature of bacteria pertaining to the family *Halanaerobiaceae* (*e.g. Halanaerobium* genus)^7^, which have been retrieved only by molecular approaches during the course of this study. In this respect, the novel isolated halophilic anaerobe^37^ might have been a microbial remnant of the original microbial community in the sedimentary basin. Finally, we demonstrate here that studying the microbiology of deep subsurface cores may be of geobiological significance by delivering important information on the existing microbial diversity at surface several millions years ago.

## Methods

### Core handling

A core was sampled within aluminum sleeves at 1153–1554 m depth below surface and immediately frozen (−80 °C) on the rigsite with liquid nitrogen. Reservoir pressure and temperature were respectively 12 Mpa and 43 °C. The core was defrosted in an anaerobic box glove chamber to ensure that no oxygen could impede development of anaerobic microorganisms. The chamber was decontaminated (bactericide and ethanol) and only sterilized materials or materials cleaned with a bactericide were introduced in order to preserve the core from any contamination during the sub-samples preparation. Only the inner part of the core (excluding ca. 2 cm in a 10 cm diameter core) was analyzed to avoid contamination by the drilling fluid. The full preservation of such unconsolidated sandstone witnesses the lack of drilling fluid invasion in the core.

### Petrographical and chemical analyses

Samples were observed under binocular for macroscopic observation, and with SEM (Philips) for ultramicroscopic analysis. X-ray diffraction was performed on a Philips θ–2θ (PW1050/81, PW3710) diffractometer. Clays were separated from the bulk sample by ultrasonication after hydrogen peroxide oxidization and grains (quartz-felspar) sedimentation.

Porosity and permeability of the sandstone were respectively between 24% and 32% and 1 to 3.5 Darcy. The water content was 12.9%. The fluid had an alkaline pH value of 8.8. Organic carbon, measured on a Flash EA CHNS/O analyzer (Thermo-scientific), was 0.38%. Cations (Ca^2+^, Na^+^, K^+^, Mg^2+^) and metals (Fe, Mn, Co, Cu, Pb, Zn) were measured by ICP-AES, Jobin Yvon JY2000 Ultrace after mineralization of the sample. Anions (Cl^−^, NO_3_^−^, SO_4_^2−^) were determined by ionic chromatography Dionex DX100. Phosphorus was measured by the Joret-Hébert method (NF X31 161), which consists of an extraction of phosphorus with oxalate before optical reading at 825 nm. Chemical analyses on the core material are given in the supplementary material Table S1.

### Dna extraction

A portion of the sediment was washed with the aim to remove hydrocarbons and PCR inhibitory material and improve DNA recovery and efficiency of PCR amplification^62^. Washing was carried out by re-suspension in wash solution (2 mL per g of sample), vortexing for 2 min, followed by centrifugation at full speed for 5 min in a bench top microfuge (14,000 g). Three successive washes were performed in wash solution 1 (50 mM Tris–HCl pH 8.3, 200 mM NaCl, 5 mM Na_2_EDTA, 0.05% Triton X-100), then in wash solution 2 (50 mM Tris–HCl pH 8.3, 200 mM NaCl, 5 mM Na_2_EDTA), and finally in wash solution 3 (50 mM Tris–HCl pH 8.3, 0.1 mM Na_2_EDTA). Despite there can be some potential loss of cell during these washing steps, this procedure was performed as it provided greater DNA quantities (data not shown). Bacterial genomic DNA was extracted from the washed sediment with the Fast DNA Spin Kit for Soil (Bio101) and the UltraClean Mega Soil DNA isolation kit (MoBio). The manufacturers protocols were slightly modified: as samples contained *a priori* low biomass, Poly-dIdC (polydexoyinosinic-deoxycytidylic acid, Sigma), a synthetic nucleotide acting both as a blocking- and carrier-agent was added at the first step of the DNA extraction procedure^63^. DNA extracts were pooled and concentrated for subsequent PCR experiments.

For identifying members of the *Bacteria* domain, 16S rRNA genes were amplified with bacterial primer 8F (5′-AGAGTTTGATCMTGGCTCAG-3′) and universal primer 1406R (5′- GACGGGCGGTGTGTRCA-3′), 30 cycles and hybridization at 55 °C. For identifying *Archaea* members, domain specific primers Arch363F (5′-ACGGGGYGCAGCAGGCGCGA-3′) and Arch915R (5′-GTGCTCCCCCGCCAATTCCT-3′), 35 cycles and hybridization at 65 °C were used.

### Clone libraries generation and screening

16S rRNA gene libraries were constructed from gel-purified (Nucleospin extract II, Macherey-Nagel) PCR products using the TOPO-TA Cloning Kit for Sequencing (Invitrogen). The gene libraries were screened for correct-length inserts by direct PCR amplification from a colony, using primers T3 and T7 targeting the plasmid. The V3 region of the insert of 92 *Bacteria* clones and 36 *Archaea* clones was then amplified respectively with primers w49/w34FAM or w36/w34FAM using the amplified inserts as templates, and grouped by identical CE-SSCP migration pattern, *i.e.* identical sequence. Selected plasmids were purified (Nucleospin Multi-8 Plus, Macherey-Nagel) and the insert was sequenced (Sanger sequencing) by Genome Express (France) with primers T7 and T3. OTU were determined by identical CE-SSCP pattern or 98% 16S rRNA gene sequence similarity.

### Sequence analyses

Sequence manipulations, analyses and alignments were performed using the BioEdit program (http://www.mbio.ncsu.edu/BioEdit/bioedit.html). Similarities of concensus 16S rRNA gene nucleotide sequences with sequences available in the Genbank database were determined using the BLASTN program (www3.ncbi.nlm.nih.gov/BLAST).

### Amplicon preparation for next-generation sequencing

Amplicons of the 16S rRNA gene suitable for sequencing on a MiSeq (Illumina) v3 kit (2 × 300bp) were produced using a nested PCR approach. During the first PCR reaction the nearly full length 16S RNA gene was amplified using the bacterial primers 8F (5′-AGAGTTTGATYMTGGCTCAG-3′) and 1492R (5′-TACCTTGTTAYGACTT-3′) for 30 cycles and hybridization at 52 °C. This step was then followed by a second PCR round using the universal primer pair 515F (5′-GTGNCAGCMGCCGCGGTAA-3′) and 926R (5′-CCGYCAATTYMTTTRAGTTT-3′) for 20 cycles and hybridization at 55 °C. The produced 440 bp long amplicons were then subjected to a third PCR amplification round using the 515MiSeq.F (5′-TCGTCGGCAGCGTCAGATGTGTATAAGAGACAGGTGNCAGCMGCCGCGGTAA-3′) and 926MiSeq.R (5′-GTCTCGTGGGCTCGGAGATGTGTATAAGAGACAGCCGYCAATTYMTTTRAGTTT-3′) primer pair which introduced Illumina’s overhang adaptors. Using Illumina’s Nextera Index kit, a final round of PCR amplification was then performed to introduce the indexes and remaining part of the Illumina’s adaptors according to the manufacturer’s recommendations.

The produced amplicons were cleaned up using the AMPure XP beads (Agencourt) and quantified on a Qubit fluorometer (Life Technologies) prior to pooling and sequencing on a MiSeq instrument (Illumina).

### Sequence analysis of miseq reads

The produced 183,776 paired-end reads were quality filtered, pair-end joined, demultiplexed and analyzed using the QIIME 1.8.0 package^64^. OTUs were picked using the uclast method^65^ at the 97% threshold. Taxonomic assignments were performed against the Greengenes database (May 2013 version).

### Cultivation

A basal medium was defined (see supplementary Table S4) and additives were added to isolate various physiological groups possibly thriving in such extreme habitats, *e.g.* yeast extract, biotrypcase and glucose for isolating fermentative microorganisms; Na_2_SO_4_ and H_2_/CO_2_ gas phase for isolating hydrogenotrophic sulfate reducers; Na_2_SO_4_ and lactate for isolating sulfate reducers oxidizing lactate; succinate and NO_3_^−^ for isolating nitrate reducers; TMA (trimethylamine) or methanol for isolating methylotrophic methanogens. Media were also designed to isolate anaerobic sulfide-oxidizers and aerobic thiosulfate oxidizers. With the first results of molecular diversity becoming available, aerobic enrichments from core samples were also performed for isolation of aerobic and anaerobic halophilic microorganisms (see supplementary Tables S5–S7). Media sterilized by filtration, and not autoclaving were also used, to prevent Maillard reaction that could impede prokaryotic development (see supplementary Table S6). pH was fixed at 8.8 (pH measured from the sediment), but 7.5, a close neutral value, was also tested, as both alkaline pH and highly saline conditions may limit growth and even survival of anaerobic micro-organisms. Temperature was fixed at 43 °C (*in situ* temperature).

### Exclusion of contamination

Several criteria can be used to evaluate if an isolate or a 16S rRNA gene sequence can be considered as native to the formation of interest or not. This includes the sampling technique as discussed above. For instance, production waters collected from separators and wellhead samples have a higher probability to contain exogenous contamination than core samples^6^. Coring was performed prior to any fluid injection in the oil field, which prevented contamination by any anthropogenic fluid. Specifically designed techniques for coring in soft materials and adapted to integrity preservation were used (aluminum sleeves, adequate drilling parameters and fluid pressure). Moreover, freezing of the core on the rigsite in liquid nitrogen will have prevented further chance of chemical and microbiological contamination. Microscopic and SEM observations of the core did not show any physical evidence (disruption, mineralogical contamination) of penetration of the drilling fluid into the core. As the clays from core samples were green and not oxidized, it seems that coring processes did not affect physico-chemical conditions of the reservoir. Only the inner part (6 cm) of the core (10 cm) was inoculated into culture media or submitted to DNA extraction. Community structures (molecular CE-SSCP fingerprints of 16S rRNA genes, data not shown) of inner and outer (2–3 cm) compartments were highly similar, supporting the absence of detectable exogenous microbe contamination by the drilling fluid. Furthermore, microbes retrieved from the molecular surveys were related to organisms showing physiological features compatible to *in situ* conditions. Another possibility to evaluate the contamination risk is to compare physiological adaptation of isolated microorganisms to the *in situ* physico-chemical conditions. The optimum temperature for growth of a microorganism isolated from an oil reservoir can be a good indicator of its physiological adaptation to the environment if it corresponds to the *in situ* temperature. In high saline reservoirs, comparison of salt tolerance or dependance of isolates can be compared to *in situ* water salinity as well. Culture optimal conditions (temperature 25–47 °C; NaCl 10–26%) of a strain isolated from the sediment and closely affiliated to the *Halobacteroides* genus were representative of the *in situ* conditions. All the halophilic microorganisms that we have isolated require subsequent concentrations of NaCl for growing, lowering the probability of microbial contamination during sample handling.

## Additional Information

**How to cite this article**: Gales, G. *et al.* Preservation of ancestral Cretaceous microflora recovered from a hypersaline oil reservoir. *Sci. Rep.* **6**, 22960; doi: 10.1038/srep22960 (2016).

## Supplementary Material

Supplementary Information

## Figures and Tables

**Table 1:** Diversity of *Archaea* and *Bacteria* retrieved in the core by 16S rRNA gene surveys.

Clone	% in library	Closest related microorganism (% of similarity by blast)	Lineage	Main metabolic traits of the related microorganisms
Members of the *Bacteria* domain - *Proteobacteria*				
MIG-B2	50.0	*Ochrobactrum* sp. (99)	*Rhizobiales; Brucellaceae*	Aerobic, mesophilic, possible degradation of crude oil by some *Ochrobactrum* spp.
MIG-B13	7.6	*Halomonas venusta* (99)	*Oceanospirillales; Halomonadaceae*	Facultative anaerobic, halophilic, marine bacterium; possible degradation of crude oil by some *Halomonas* spp.
MIG-B23	4.3	*Halomonas meridiana* (99) *Halomonas aquamarina* (99)	*Oceanospirillales; Halomonadaceae*	Facultative anaerobic, halophilic, marine bacterium; possible degradation of crude oil by some *Halomonas* spp.
MIG-B19	4.3	*Burkholderia ferrariae* (99) *Burkholderia silvatlantica* (99)	*Burkholderiales; Burkholderiaceae*	Aerobic, mesophilic; possible degradation of alkanes and aromatic hydrocarbons by some *Burkholderia* spp.
MIG-B18	2.2	*Rhizobium loti* (99)	*Rhizobiales; Phyllobacteriaceae*	Aerobic and mesophilic bacterium
MIG-B1	1.1	*Halomonas phoceae* (97) *Halomonas xinjiangensis* (97)	*Oceanospirillales; Halomonadaceae*	Facultative anaerobic, halophilic, marine bacterium; possible degradation of crude oil by some *Halomonas* spp.
MIG-B9	1.1	*Chromohalobacter salexigens* (99)	*Oceanospirillales; Halomonadaceae*	Aerobic, moderate halophilic, marine bacterium
Members of the *Bacteria* domain – *Firmicutes*, *Actinobacteria*, and *Thermotogae*				
MIG-B16	19.6	*Halolactibacillus halophilus* (98) *Halolactibacillus miuriensis* (98)	*Firmicute; Bacillales; Bacillaceae*	Facultative anaerobic, halophilic and alkaliphilic marine lactic acid bacterium
MIG-B8	4.3	*Halanaerobium fermentans* (99)	*Firmicute; Halanaerobiales; Halanaerobiaceae*	Anaerobic, halophilic fermentative bacterium
MIG-B25	2.2	*Orenia marismortui* (95)	*Firmicute; Halanaerobiales; Halobacteroidaceae*	Anaerobic, halophilic fermentative bacterium
MIG-B42	1.1	*Orenia marismortui* (95)	*Firmicute; Halanaerobiales; Halobacteroidaceae*	Anaerobic, halophilic fermentative bacterium
MIG-B15	1.1	*Microbacterium paraoxydans* (99)	*Actinobacteria Actinomycetales; Microbacteriaceae*	Aerobic, mesophilic; possible degradation of crude oil by some *Microbacterium* spp.
MIG-B3	1.1	*Geotoga aestuarianus* (99)	*Thermotogales; Thermotogaceae*	Anaerobic, thermophilic fermentative bacterium
Members of the *Archaea* domain - *Euryarchaeota*				
MIG-ARCH1	33.3	*Halococcus hamelinensis* (99)	*Halobacteriales*	Aerobic, extreme halophile
MIG-ARCH6	30.6	*Haloplanus natans* (98)	*Halobacteriales*	Aerobic, extreme halophile
MIG- ARCH2	16.7	*Halorhabdus tiamatea* (97) *Halorhabdus utahensis* (97)	*Halobacteriales*	Facultative anaerobic (microaerobic) extreme halophile
MIG-ARCH27	16.7	*Halarchaeum acidiphilum* (97)	*Halobacteriales*	Aerobic, extreme halophile
MIG-ARCH10	2.8	*Thermococcaceae* (77)	*Thermococcales*	Anaerobic hyperthermophile

Retrieved environmental 16S rRNA gene sequences are available under Genbank accession numbers JQ690672 to JQ690688.

**Table 2:** Diversity of *Bacteria* retrieved from the core using Illumina sequencing of the 16S rRNA gene.

% in library	Closest relative*	Lineage	Main metabolic traits of the related microorganisms
27.9	*Halomonas*	*Gammaproteobacteria; Oceanospirillales; Halomonadaceae*	Facultative anaerobic, halophilic, marine bacterium; possible degradation of crude oil by some *Halomonas* spp.
11.0	*Desulfonauticus*	*Deltaproteobacteria; Desulfovibrionales; Desulfohalobiaceae*	Anaerobic, thermophilic sulfate-reducing bacteria isolated from oil-production water and deep-sea hydrothermal vents
10.1	*Pseudomonas*	*Gammaproteobacteria; Pseudomonadales; Pseudomonadaceae*	Aerobic, known to degrade aromatic hydrocarbons
7.1	*Marinobacter*	*Gammaproteobacteria; Alteromonadales; Alteromonadaceae*	Facultative anaerobic, halophilic, marine bacterium; possible degradation of crude oil by some *Marinobacter* spp.
6.1	n.d.	*Gammaproteobacteria; Enterobacteriales; Enterobacteriaceae*	Facultative anaerobic, many of them are nitrate reducers under anaerobic conditions
5.7	n.d.	*Bacteroidia; Bacteroidales;*	
4.2	n.d.	*Clostridia; Clostridiales; Clostridiaceae*	Primarily anaerobic; many of its genera are fermenters
2.9	n.d.	*Deltaproteobacteria; Desulfovibrionales;*	Obligatory anaerobic, mesophilic or moderately thermophilic sulfate reducers
2.1	n.d.	*[Cloacamonae]; [Cloacamonales]; MSBL8*	Affiliated with *Spirochaetes*, anaerobic, halotolerant
1.4	*Oceanobacillus*	*Bacilli; Bacillales; Bacillaceae*	Halotolerant, alkaliphilic, mesophilic, facultative anaerobic or strictly aerobic bacterium
1.2	n.d.	*Gammaproteobacteria; Oceanospirillales; Oceanospirillaceae*	Most genera are halophilic, aerobic or facultative anaerobic, chemoorganotrophs
1.1	n.d.	*Betaproteobacteria; Burkholderiales; Comamonadaceae*	Aerobic organotrophs, anaerobic denitrifiers and Fe(III)-reducing bacteria, hydrogen oxidizers, photoautotrophic and photoheterotrophic bacteria, and fermentative bacteria, members are known to degrade hydrocarbons
0.9	*Ralstonia*	*Betaproteobacteria; Burkholderiales; Oxalobacteraceae*	Aerobic, can reduce nitrate anaerobically, found in both soil and water, used in bioremediation, chlorinated aromatic compounds degrader
0.8	*Desulfovibrio*	*Deltaproteobacteria; Desulfovibrionales; Desulfovibrionaceae*	Anaerobic, sulfate-reducer
0.7	*Pelobacter*	*Deltaproteobacteria; Desulfuromonadales; Pelobacteraceae*	Strictly anaerobic, Fe(III) or S(0) reducer
0.6	*Halanaerobium*	*Clostridia; Halanaerobiales; Halanaerobiaceae*	Anaerobic, halophilic fermentative bacterium
0.4	n.d.	*Flavobacteria; Flavobacteriales; Flavobacteriaceae*	
0.4	*Thermovirga*	*Synergistia; Synergistales; Thermovirgaceae*	Moderately thermophilic, anaerobic, sulfur reducing, amino-acid-degrading bacterium
0.3	*Marinilactibacillus*	*Bacilli; Lactobacillales; Aerococcaceae*	Facultative anaerobic, halophilic, alkaliphilic, marine, mesophilic, lactic acid producing bacterium
6.2	Unassigned		
9.0	Other		

Sequences are available at BioProject ID PRJNA301727; n.d.: not determined.

**Table 3:** Microbial diversity of isolates originating from the hypersaline oil reservoir and their condition of isolation.

Isolates	Total	Closest phylogenetic relative	% blast similarity	Condition of isolation
RAH4 and FERM001	2	*Thalassobacillus devorans*	99–100	250 g.l^−1^ NaCl pH 8.8 anaerobiosis (Table S2)
FERM003 and AER001	2	*Bacillus circulans*	99–100	120 g.l^−1^ NaCl pH 8.8 anaerobiosis (Table S2)
1D4	1	*Orenia marismortui*	99	250 g.l^−1^ NaCl pH 8.8 anaerobiosis (Table S2)
Delta1, Delta2 and Delta3	3	*Halanaerocella petrolearia* (Strain S200^T^)	100	250 g.l^−1^ NaCl pH 8.8 anaerobiosis (Tables S3 and S6)
Alpha	1	*Halobacillus trueperi*/*dabanensis*	99	250 g.l^−1^ NaCl pH 7.5 anaerobiosis (Table S2)

16S rRNA gene sequences of isolates are available under Genbank accession numbers JQ690689 to JQ690697.
