# Supplementary material for: Preservation of ancestral Cretaceous microflora recovered from a hypersaline oil reservoir
Source: Sci Rep. 2016 Mar 11;6:22960. doi: 10.1038/srep22960 (PMC4786803; doi:10.1038/srep22960)
Supplement: Supplementary Information [file srep22960-s1.doc]

**Preservation of ancestral Cretaceous microflora recovered from a hypersaline oil reservoir - Supplementary material**

Grégoire Gales, Nicolas Tsesmetzis, Isabel Neria, Didier Alazard, Stéphanie Coulon, Bart P. Lomans, Dominique Morin, Bernard Ollivier, Jean Borgomano & Catherine Joulian

**Supplementary Table S1.** Chemical analyses performed on the core material

| Chemical | Concentration | Standard deviation |
| --- | --- | --- |
| Ca^2+^ | 3.6 mg/g | 0.2 mg/g |
| Na^+^ | 13.1 mg/g | 0.9 mg/g |
| K^+^ | 13.8 mg/g | 1.0 mg/g |
| Mg^2+^ | 5.1 mg/g | 0.3 mg/g |
| Fe | 3907 µg/g | 203 µg/g |
| Mn | 33.7 µg/g | 0.8 µg/g |
| Co | 154.6 µg/g | 1.6 µg/g |
| Cu | 115.8 µg/g | 11 µg/g |
| Pb | - | - |
| Zn | 9.1 µg/g | 1.1 µg/g |
| NH_4_^+^ | 13.65 µg/g | 0.64 µg/g |
| Cl^-^ | 2.71 mg/g | 0.2 mg/g |
| NO_3_^-^ | 0.18 mg/g | 0.03 mg/g |
| SO_4_^2-^ | 0.44 mg/g | 0.04 mg/g |
| P_2_O_5_ | 7,1 µg/g | 2 µg/g |
| Organic carbon | 0.38 % | 0.04 % |

**Supplementary Figure S2.** Rarefaction curves of bacterial and archaeal 16S rRNA gene sequences retrieved from the saline oil reservoir core. OTU were determined identical CE-SSCP pattern or 98% 16S rRNA gene sequence similarity. Curves were obtained using the EcoSim software freely available at <http://www.garyentsminger.com/ecosim/index.htm>.


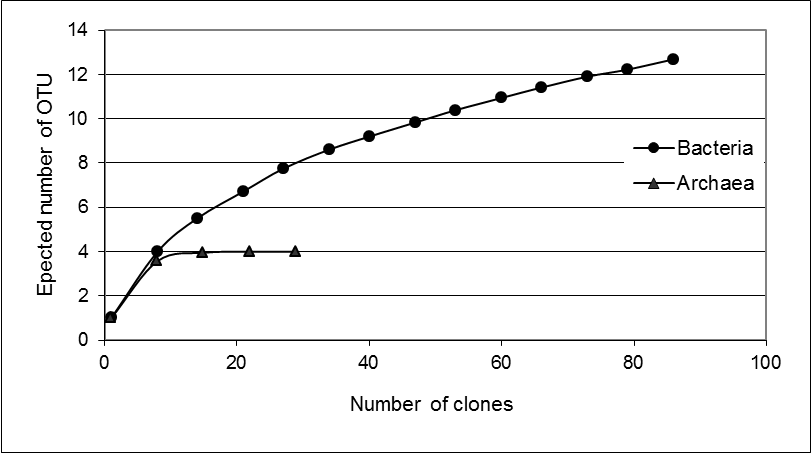


**Supplemental information about the cultivation procedure**

The composition of the basal medium used in this study is given below (Table S2). MgCl_2_.6H_2_O concentration was fixed at a 1/10^th^ of the NaCl concentration. Depending on the target metabolisms, the following additives were supplemented from stock anaerobic solutions to the sterile basal medium: for sulfate reducers, 20 mM of Na_2_SO_4_, and 1 bar of H_2_/CO_2_ (v/v) or 20 mM lactate; for nitrate reducers, 10 mM KNO_3_ and 10 mM Na succinate; for methanogens, 10 mM trimethylamine or 10 mM methanol; for fermentative prokaryotes, 5 g.l^-1^ yeast extract, 5 g.l^-1^ biotrypcase and 20 mM glucose, aerobic conditions being also tested; for sulfide and thiosulfate oxidizers, 1 mM sulfide or 20 mM sodium thiosulfate with 10 mM KNO_3_ or aerobic conditions. For each condition, the pH was fixed at 8.8 (pH measured from the sediment) or 7.5; we tested a close neutral value of pH, as both alkaline pH and highly saline conditions may limit growth and even survival of anaerobic micro-organisms.

Regarding 16S rRNA gene sequences encountered by molecular techniques, new culture media and culture conditions (e.g. pH, salt concentration, use of antibiotics, anaerobiosis/aerobiosis) were defined for isolation of aerobic and anaerobic halophilic microorganisms (Tables S3 to S7), as most of anaerobic Halobacteria are considered as moderately halophiles, whereas aerobic Haloarchaea are considered as extremely halophilic.

All isolation procedures were performed using the same protocol. For anaerobic cultures, sterile Hungate tubes and serum bottles sealed under an O_2_-free N_2_ atmosphere were used. First, Rabi sand (15%, w/v) was inoculated in the different liquid culture media. Second, after growth observation, roll-tubes (anaerobic conditions) or Petri dishes (aerobic conditions) were used to isolate colonies on solid medium. Third, restrictive dilutions of single colonies were made in the same liquid medium. Finally, inoculation of the last growing dilution was performed in four tubes filled with same liquid medium. Three of them were used for DNA extraction, whereas the fourth one was used as a strain deposit in our collection of microorganisms for further studies, if necessary.

All cultures were performed at 43°C.

**Supplementary Table S4:** Basal medium for cultivation of anaerobic sulfate-reducing, nitrate-reducing, methanogenic and fermentative Prokaryotes

| Component | Concentration (g.l^-1^) |
| --- | --- |
| NaCl | 120 / 250 |
| Yeast extract | 0.1 |
| Tryptone | 0.5 |
| Cysteine-HCl | 0.5 |
| K_2_HPO_4_ | 0.3 |
| KH_2_PO_4_ | 0.3 |
| NH_4_Cl | 1 |
| KCl | 0.1 |
| MgCl_2_.6H_2_O | 12 / 25 |
| CaCl_2_.2 H _2_O | 0.1 |
| FeCl_2_.4H_2_O | 0.036 |
| MnCl_2_.6H_2_O | 0.00036 |
| Trace element solution* | 1 ml |

* Widdel, F. & Pfennig, N. Studies on dissimilatory sulfate-reducing bacteria that decompose fatty acids. I. Isolation of new sulfate-reducing bacteria enriched with acetate from saline environments. Description *of Desulfobacter postgatei* gen. nov., sp. nov. *Arch. Microbiol.* **129,** 395-400 (1981).

Before inoculation (5 ml culture medium), 0.1 ml 10% NaHCO_3_, 0.1 ml 2% Na_2_S were injected from sterile stock solutions.

**Supplementary Table S5:** Medium defined for the cultivation of fermentative Prokaryotes

| Component | Concentration (g.l^-1^) |
| --- | --- |
| NH_4_Cl | 1 |
| K_2_HPO_4_ | 0.3 |
| KH_2_PO_4_ | 0.3 |
| MgCl_2_.6H_2_O | 10 |
| MgSO_4_.7H_2_O | 5 |
| CaCl_2_.2 H _2_O | 0.1 |
| NaCl | 250 |
| KCl | 0.1 |
| Yeast extract | 1 |
| Trace element solution* | 10 ml |

* Balch, W.E., Fox, G.E., Magrum, R.J. & Wolfe, R.S. Methanogens: reevaluation of a unique biological group. *Microbiol Rev* **43**:260–296 (1979).

pH was fixed at 7.5 or 8.8.

Before inoculation (5 ml culture medium), 0.1 ml 10% NaHCO_3_, 0.1 ml 2% Na_2_S, and 0.1 ml glucose (1 M) were injected from sterile stock solutions.

**Supplementary Table S6:** Medium for cultivation of aerobic and anaerobic haloalkaliphilic Prokaryotes

| Component | Concentration (g.l^-1^) | | Concentration (g.l^-1^) | |
| --- | --- | --- | --- | --- |
| NaCl | | 100 / 200 | | None |
| Sea salts | | None | | 30 / 150 |
| Yeast extract | | 5 | | 5 |
| Peptone | | 5 | | 5 |
| Sodium glutamate | | 1 | | 1 |
| Trisodium citrate | | 3 | | 5 |
| MgSO_4_.7H_2_O | | 10 | | None |
| KCl | | 2 | | None |
| FeCl_2_.4H_2_O | | 0.036 | | None |
| MnCl_2_.6H_2_O | | 0.00036 | | None |
| pH | | 8.8 | | 8.8 |
|  | | Aerobic | | Anaerobic |

pH was fixed at 8.8 or 7.5.

***Supplementary Table S7:*** *Medium for cultivation of halophilic aerobic and anaerobic* Archaea

| Component | Concentration (g.l^-1^) | Concentration (g.l^-1^) |
| --- | --- | --- |
| NaCl | 200 | None |
| Sea salt | None | 250 |
| Yeast extraction | 10 | 10 |
| Sodium glutamate | 1 | 1 |
| Casamino acids | 10 | 10 |
| Glucose | 2 | 2 |
| Maltose | 2 | 2 |
| MgSO_4_.7H_2_O | 10 | None |
| KCl | 2 | None |
| FeCl_2_.4H_2_O | 0.036 | None |
| MnCl_2_.6H_2_O | 0.00036 | None |

For each medium (*e.g.* with NaCl or sea salt), we tested the influence of aerobiosis or anaerobiosis, of the presence of 0.2 or 5 mL of antibiotics solution (per mL: chloramphenicol, 100 ng ; tretracycline, 100 ng ; streptomycine, 200 ng. Sterilization was performed by 0.2 µm filtering.)

The pH was fixed at 8.8 or 7.5.

**Supplementary Table S6:** Medium for cultivation of members of the *Haloanaerobiales* (Haloanaerobes)

| Component | Concentration (g.l^-1^) | Concentration (g.l^-1^) |
| --- | --- | --- |
| NaCl | 100 / 200 | None |
| Sea salt | None | 100 / 200 |
| Yeast extract | 1 | 1 |
| Biotrypcase | 1 | 1 |
| Glucose | 10 | 10 |
| Sodium acetate | 0.9 | 0.9 |
| NH_4_Cl | 1 | 1 |
| K_2_HPO_4_ | 0.3 | 0.3 |
| KH_2_PO_4_ | 0.3 | 0.3 |
| MgCL_2_.6H_2_O | 1 / 2 | None |
| KCl | 2 / 4 | None |
| CaCl_2_.2H_2_O | 1 / 2 | None |
| Resazurine | 1 | 1 |
| Trace element solution* (ml) | 1 | 1 |

* Widdel, F. & Pfennig, N. Studies on dissimilatory sulfate-reducing bacteria that decompose fatty acids. I. Isolation of new sulfate-reducing bacteria enriched with acetate from saline environments. Description *of Desulfobacter postgatei* gen. nov., sp. nov. *Arch. Microbiol.* **129,** 395-400 (1981).

pH was fixed at 8.8 or 7.5.

**Supplementary Table S7:** Medium without autoclaving cycle (sterilized by filtration to avoid Maillard reactions during autoclave cycles, which generate toxic compounds and may therefore impede microbial growth)

| Component | Concentration (g.l^-1^) |
| --- | --- |
| NaCl | 200 / 250 |
| Yeast extract | 5 |
| Sodium glutamate | 1 |
| Trisodium citrate | 3 |
| Pyruvate | 1 |
| NH_4_Cl | 0.2 |
| CaCl_2_.2H_2_O | 0.1 |
| KCl | 2 |
| MgCL_2_.6H_2_O | 2 / 20 |
| FeCl_2_.4H_2_O | 0.036 |
| MnCl_2_.6H_2_O | 0.00036 |

pH was fixed at 8.8 and 7.5.
